# Chemotherapy and Chemoprevention by Thiazolidinediones

**DOI:** 10.1155/2015/845340

**Published:** 2015-03-19

**Authors:** Eleonore Fröhlich, Richard Wahl

**Affiliations:** ^1^Internal Medicine, Department of Endocrinology, Metabolism, Nephrology and Clinical Chemistry, Department IV, University of Tuebingen, Otfried-Muellerstrasse 10, 72076 Tuebingen, Germany; ^2^Center for Medical Research, Medical University of Graz, Stiftingtalstraße 24, 8010 Graz, Austria

## Abstract

Thiazolidinediones (TZDs) are synthetic ligands of Peroxisome-Proliferator-Activated Receptor gamma (PPAR*γ*). Troglitazone, rosiglitazone, and pioglitazone have been approved for treatment of diabetes mellitus type II. All three compounds, together with the first TZD ciglitazone, also showed an antitumor effect in preclinical studies and a beneficial effect in some clinical trials. This review summarizes hypotheses on the role of PPAR*γ* in tumors, on cellular targets of TZDs, antitumor effects of monotherapy and of TZDs in combination with other compounds, with a focus on their role in the treatment of differentiated thyroid carcinoma. The results of chemopreventive effects of TZDs are also considered. Existing data suggest that the action of TZDs is highly complex and that actions do not correlate with cellular PPAR*γ* expression status. Effects are cell-, species-, and compound-specific and concentration-dependent. Data from human trials suggest the efficacy of TZDs as monotherapy in prostate cancer and glioma and as chemopreventive agent in colon, lung, and breast cancer. TZDs in combination with other therapies might increase antitumor effects in thyroid cancer, soft tissue sarcoma, and melanoma.

## 1. Introduction

**Figure 1:**
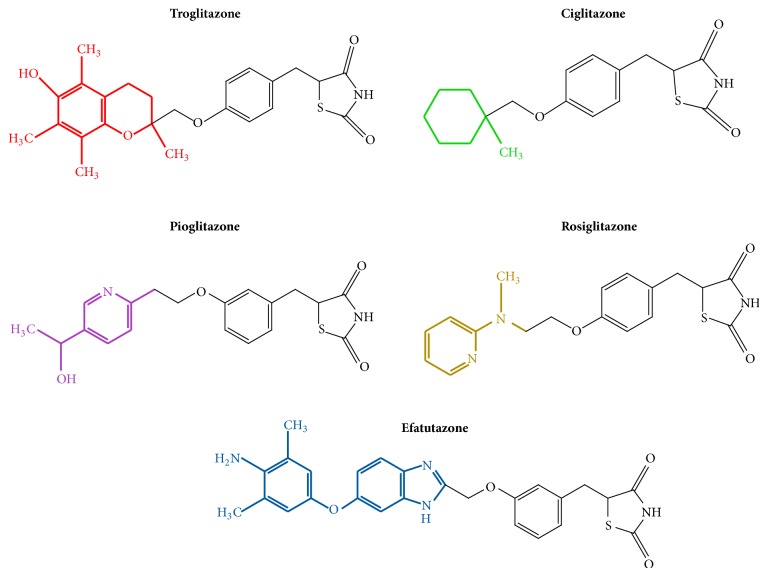
Chemical formulae of the most common TZDs with antitumor action.

Glitazones, also called thiazolidinediones (TZDs), are five-membered carbon ring molecules containing two heteroatoms (nitrogen and sulfur). One carbonyl group in the thiazole at position 4 and another at position 2 make the heterocyclic compound a thiazolidine-2,4-dione [1]. TZDs are ligands of the Peroxisome Proliferator Activated Receptor gamma (PPAR*γ*), a nuclear receptor inducing upregulation of specific genes that decrease insulin resistance, inflammation, VEGF-induced angiogenesis, proliferation, and leptin levels, inducing differentiation of adipocytes, and increasing adiponectin levels. This spectrum of actions led to the approval of TZDs for treatment of diabetes mellitus type II. TZDs differ according to the substitution at C5 (Figure 1).

Ciglitazone (CIGLI) is the prototype of all TZDs but has never been approved for medication of diabetes mellitus because its clinical activity was too weak. Troglitazone (TRO) was the first TZD approved for treatment of diabetes mellitus in 1997 [2]. The compound showed beneficial effects on glucose levels, insulin sensitivity, and free fatty acid concentration but was withdrawn from the market in 2000 due to severe hepatotoxicity. The second TZD, rosiglitazone (ROSI), has been banned in Europe and restricted in the USA because of increased cardiovascular morbidity. Also the use of pioglitazone (PIO) as the third TZD with antidiabetic action is restricted due to concerns about a potential facilitation of bladder cancer development. The fourth substance with an antidiabetic profile, rivoglitazone, is still under investigation [3]. Reasons for the troubled history of antidiabetic TZDs are manifold and appear to be due to the highly pleiotropic action of these PPAR*γ* agonists and crosstalk of PPAR*γ* with other signaling pathways.

In addition to diabetes mellitus treatment, ligands to PPAR*γ* could also be exploited for treating other diseases, for instance, in cancer treatment. This idea originated from the finding that PPAR*γ* is involved in cell proliferation and PPAR*γ* expression levels change from normal to transformed tissues. Effects of PPAR*γ* activation are ligand-specific. TZDs with potent PPAR*γ* agonist activity can display, like rivoglitazone, strong antidiabetic activity, or, like efatutazone (EFA), predominantly antitumor effects. TZDs, such as netoglitazone, can also activate other PPARs and cause antitumor effects [4]. This review will focus on the effects of selective PPAR*γ* TZDs in tumors.

## 2. Role of PPAR***γ*** Expression in Neoplasms

PPAR*γ* expression compared to normal tissue tends to be increased in precursor lesions and differentiated tumors and decreased in the poorly differentiated cancers. This pattern has been reported for instance for gastric, ureteric, and breast cancer [5–7]. In ovarian cancer, however, PPAR*γ* levels independent from tumor differentiation are increased [8]. Upregulation of PPAR*γ* may be an early event in tumorigenesis and a marker for differentiated cancer lesions [9]. Methylation (silencing) of the PPAR*γ* promotor, which is detected in 30% of colorectal tumors, however, correlated with poor prognosis [10]. Studies linking tumor prognosis and PPAR*γ* expression were mainly based on immunohistochemical detection of the PPAR*γ* antigen in paraffin-embedded tissue. Since antigenicity is low and may decrease during storage of the paraffin samples, the absence of PPAR*γ* staining in archival tissues may be a false negative due to methodological problems [11].

Identification of the contribution of PPAR*γ* to tumor development and progression is further complicated by crosstalk with other pathways. Akt phosphorylation in the endometrium, for instance, is directly regulated by PPAR*γ* and indirectly through induction of PTEN by PPAR*γ*, where PTEN decreases p-Akt via inhibition of PI3K [12].

## 3. Mechanism of Antitumor Action by TZDs

Although all TZDs are PPAR*γ* ligands, the observed antitumor effects can only be explained in part by genomic PPAR*γ* activation. Genomic activation is defined as the binding of a nuclear receptor to a response element, which activates the transcription of certain genes. The process is also termed transactivation. Another DNA-mediated effect is transrepression, which describes the binding of receptors to transcription factors (e.g., nuclear factor kappa B (NF*κ*B) or activator protein 1 (AP-1)).

**Figure 2:**
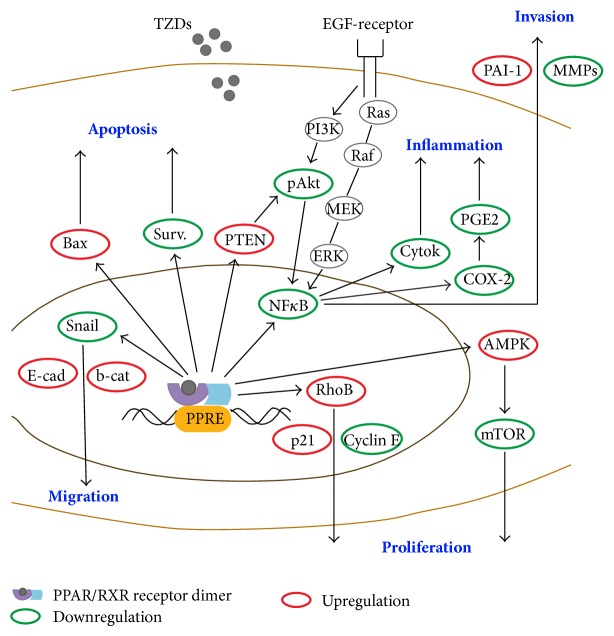
Effects of TZDs on apoptosis, migration, invasion, and proliferation of cancer cells and on inflammation. In some ellipses, only one representative is listed; Bax and p53 react similarly, as well as p27 and p21. MMPs represents MMP-2 and MMP-9 and Cyclin E represents cyclin D1, cyclin B1, CDK2, and CDK4. Abbreviations: EGF: epithelial growth factor receptor; PPRE: PPAR*γ* response element, Surv: survivin, E-cad: E-cadherin, b-cat: *β*-catenin, Cytok: cytokines.

PPAR*γ* ligands trigger a conformational change of the PPAR*γ* receptor that attracts transcriptional coactivators of the steroid receptor coactivator family. Once activated by ligand binding, the PPAR*γ* receptor forms heterodimers with the retinoid X-receptor and transcription is initiated. Transcriptional activation may result in decreased proliferation, migration and inflammation and increased differentiation and apoptosis (Figure 2). Inflammatory effects are usually mediated by transrepression [13].

Figure 2 illustrates the variety of pathways influenced by genomic activation of PPAR*γ* by TZDs, resulting in downregulation of migration, proliferation, inflammation, and invasion and in upregulation of apoptosis. Common mechanisms involve influence on EGF signaling, cyclins, Ki-67, c-myc, cyclin-dependent kinases, p53 and PTEN expression, adhesion proteins, metalloproteinases, and cytokines [14–19].

Hormone-dependent cancers react through different mechanisms to TZDs depending on the hormone receptor status. In androgen-dependent prostate carcinoma, for instance, CIGLI downregulated aromatase activity, while in androgen-independent tumors proliferation was reduced [20].

Different TZDs may act by different mechanisms; while CIGLI downregulated cyclin D1 and upregulated p21 by PPAR*γ* independent pathways, ROSI used PPAR*γ* signaling to induce these effects in androgen-independent prostate carcinoma cells [21].

The description of all mechanisms of TZDs is beyond the scope of this review but one important signaling pathway for tumor cells and for surrounding tissue (tumor microenvironment) each illustrates the variety of PPAR*γ* effects. Tumor biology is not only determined by tumor cells but to a high extent by properties of stromal cells in the tumor microenvironment. Among the diverse cells in the tumor stroma (endothelial cells, cancer-associated fibroblasts, leukocytes, myofibroblasts, and mesenchymal stem cells), tumor-associated macrophages play the most decisive role in tumor progression [22].

For tumor cells, signaling by Epidermal Growth Factor receptor (EGF-receptor, Figure 2) is highly relevant. The signaling cascade of the EGF-receptor involves the ERK cascade, consisting of Ras-Raf-MEK1/MEK2-ERK1/ERK2 and is seen in several cancer types [23]. ERK may phosphorylate PPAR*γ* and reduce its genomic activity. This effect occurs in cancer cell lines and a variety of normal cells alike [24]. TRO, for example, was reported to bind to the EGF receptor and trigger its internalization in EGF-receptor transfected endothelial cells [25]. This action is an example of nongenomic effects of TZDs since no ligand binding to response element occurred.

Normal macrophages can transform into tumor-associated macrophages under stimulation of PPAR*γ* ligands [26]. ROSI decreased activation of macrophages and thereby reduced inflammation in nondiabetic patients with symptomatic carotid artery stenosis [27]. In murine macrophages, these effects are mediated by interaction of PPAR*γ* with Nf*κ*B [28]. In these effects, transrepression appears to be the main mechanism.

Finally, MEK1 action by ROSI may lead to nuclear export and cytoplasmic retention of PPAR*γ* and off-DNA interaction with proteins in MEK1-GFP and PPAR*γ* (wild-type and mutant) cotransfected HEK-293 cells [29]. In this effect no genomic action of TZDs was involved.

## 4. Therapeutic Efficacy of TZDs in Specific Cancers

Decrease of cell proliferation, cytotoxicity, and proapoptotic effects induced by CIGLI, TRO, ROSI, and PIO has been reported in a variety of cell lines (sarcoma, melanoma, glioblastoma, breast carcinoma, colorectal cancer, gastric cancer, pancreatic cancer, prostate, bladder cancer, hepatic cancer, thyroid cancer, ovarian cancer, endometrial cancer, and lung cancer cells), which will not be listed in detail. Based on promising cellular action, animal experiments and clinical trials have been conducted in several common cancers.

EFA, which was developed as a chemostatic rather than an antidiabetic drug, has also been studied in some of these cancers. EFA is 500x more potent an activator of PPAR*γ* than TRO and 50x stronger than ROSI. EFA was studied in a preclinical murine model for breast cancer based on BRCA1 (BReast CAncer 1) deficiency. In the MMTV-Cr BRCA1^flox/flox^  p53^+/−^ model, exon 11 of the BRCA1 gene is deleted by Mouse Mammary Tumor Virus (MMTV)-Cre transgene. The deletion is accompanied by loss of one germline copy of TP53. EFA reduced the incidence of noninvasive and well-differentiated tumors in this model [30].

Cell proliferation and xenograft size of pancreatic, anaplastic thyroid, and colorectal cancer were reduced by EFA administration [31].

Based on these promising preclinical effects, phase I trials were initiated either as monotherapy or in combination with other compounds. After monotherapy with EFA, stable disease was induced in 10/22 patients with advanced liposarcoma [14]. A phase 1 study evaluating the combination of bexarotene with EFA in solid tumors is currently recruiting patients (NCT01504490).

The first trial of antitumor effects of the antidiabetic TZDs was conducted in three liposarcoma patients, where decrease of proliferation with TRO has been reported [32]. No beneficial effects, however, were obtained in a trial with ROSI in 9 liposarcoma patients [33]. Despite the negative outcome of this trial, another phase II trial on ROSI is ongoing (NCT00004180; http://www.cancer.gov/clinicaltrials/).

TZDs showed variable efficacy in studies of common cancers using xenograft and transgenic mouse models, in case studies and clinical trials (an overview is provided in Table 1).

### 4.1. Colorectal Cancer

Studies on human tumor samples support the hypothesis that PPAR*γ* expression has protective effects in colorectal cancer [34]; patients with PPAR*γ* expression usually showed a better prognosis [11]. Accordingly, reduction of *β*-catenin and PPAR*γ* was associated with high numbers of tumor-associated macrophages, increased metastasis, and poor survival [35]. On the other hand, loss of function point mutations of the PPAR*γ* gene and polymorphisms in PPAR*γ* genes were encountered in 8% of colorectal carcinoma patients, but some studies on PPAR*γ* expression in colorectal samples did not find any relation of PPAR*γ* immunoreactivity and tumor parameters [36, 37]. The role of PPAR*γ* activation in the progression of malignant lesions is questioned by the fact that heterozygous and homozygous intestinal-specific PPAR*γ* deficiency promoted tumor formation [38]. This suggests that murine models might not be representative for the study of TZDs in colorectal cancer.

Consistent with the unclear role of PPAR*γ* in tumor samples, TZDs showed variable effects* in vivo*. PPAR*γ* activation inhibited xenograft growth in mice and PPAR*γ* agonists reduced the number of aberrant cryptal foci in chemically induced inflammatory bowel disease in mice [39, 40]. On the other hand, PIO induced increased polyp numbers in mice with APC mutation, prone to developing colon adenoma (APC^min⁡^), not in wild-type mice, suggesting that, under certain genetic conditions, TZDs could also promote colon cancer development [41]. The disparate results might be explained by* in vitro* studies in colon cancer cell lines showing that the level of PPAR*γ* expression correlated to cells' sensitivity to proliferation inhibition [42].

A phase II trial with TRO did not increase progression-free survival in 25 colorectal cancer patients [43].

### 4.2. Lung Cancer

PPAR*γ* expression in well-differentiated lung adenocarcinoma was higher than in poorly differentiated tumors, suggesting that it promotes tumor formation but is not a marker for aggressive growth [44]. In another study, expression was linked to poor prognosis, showing the opposite trend [45]. ROSI decreased progression of chemically induced murine cancer model [46].

### 4.3. Breast Cancer

In breast cancer PPAR*γ* mRNA levels did not correlate with nodal involvement and tumor grade but significantly lower PPAR*γ* levels were seen in large metastatic tumors, patients with local recurrence and poor survival [47]. Despite the fact that samples of aggressive tumors showed increased PPAR*γ* expression, TZDs displayed moderate positive effects in breast cancer models. ROSI reduced tumor growth in a chemically induced rat and in a syngenic murine tumor model [48, 49]. Both in patients with advanced breast carcinoma and in patients with early mammary cancer treatment with TZDs did not cause therapeutic effects [50, 51].

### 4.4. Prostate Cancer

In the majority of prostate cancers (73%), immunoreactivity and expression of PPAR*γ* correlated inversely with tumor size and PSA levels [52]. Data obtained in prostate cancer xenografts as well as results from a phase II trial and a case report showed efficacy of PIO and TRO [53–55].

### 4.5. Glioblastoma

No correlation of PPAR*γ* expression has been established with glioma [56]. Diabetes mellitus patients under TZD medication, however, showed lower incidence of high-grade glioma than the control group (patients with hip fractures), while survival of patients with glioma was similar in both groups [57]. Efficacy of PIO has been shown in glioma xenografts and in a phase II trial [58, 59].

### 4.6. Melanoma

No correlation of PPAR*γ* expression and melanoma prognosis was seen [60]. In a cohort study of diabetes mellitus patients under PIO medication, an increased hazard ratio for melanoma (1.3) was reported [61]. It is not clear whether these data represent an increased incidence of tumors because the maximum duration of follow-up was <6 years after the initiation of PIO. Studies on monotherapy with TZDs in melanoma are limited: only CIGLI was reported to inhibit growth of melanoma xenografts [62].

Higher mRNA or protein expression in well-differentiated tumors compared to poorly differentiated tumors and tumors with poor prognosis is interpreted as protective effect of PPAR*γ* in tumor development. In prostate cancer patients, protective effects of PPAR*γ* and therapeutic effect of TZDs were in line (Table 1). In glioma samples, PPAR*γ* expression was not linked to good prognosis but TZDs showed therapeutic efficacy.

## 5. Role of TZDs in Chemoprevention

While therapeutic efficacy of monotherapy with TZDs was relatively low, data obtained from meta-analysis of diabetes studies as well as* in vitro* data suggested that TZDs could be efficient in chemoprevention (Table 2).

### 5.1. Data from Diabetes Trials

Medication with TZDs for >1 year decreased the incidence of head and neck cancers by 40% and lung cancer by 33% in diabetes mellitus patients [63]. The reduction of lung cancer reached 75% in the African-American population. The reduction was specific for lung cancer, as prostate and colorectal cancer incidence was not changed. Of note, in this study, patients with preexisting malignancies were excluded. The largest meta-analysis on cancer incidence and cancer mortality included data of 46 trials. The number of malignancies was disclosed in 28/33 trials with ROSI and in 18/33 trials with PIO [64]. This meta-analysis reported less cancer cases (342 versus 457) in patients treated with TZDs compared to other medications. Overall, treatment with TZDs was associated with a significantly lower incidence of cancer cases (Mantel-Haenszel odds ratio (MH-OR) 0.85; *P* = 0.027). For ROSI this effect was significant for colorectal cancer (MH-OR 0.63; *P* = 0.03). PIO treatment significantly reduced the incidence of breast cancer (MH-OR 0.28; *P* = 0.004). An increase in the incidence of bladder cancer by PIO treatment was not seen (MH-OR 2.05; *P* = 0.12), but cancer mortality was increased upon TZD treatment. Since this mortality most probably is due to preexisting cancers, the question remains whether treatment with TZDs could promote the growth of already existing malignant lesions.

### 5.2. *In Vitro* Differentiation Studies

Morphological differentiation (duct formation in collagen gels) increased in pancreatic carcinoma cells treated with TRO [65] and increases of villin and mucin mRNA were observed in colon cancer cell lines [66]. ROSI induced PTEN expression in Caco-2 cells and restored glandular morphogenesis [67]. It increased tyrosinase expression, an indication for differentiation, in a melanoma cell line [68]. ROSI also caused reversal of epithelial-mesenchymal transition in anaplastic thyroid cancer cell lines and increased expression of thyroglobulin, TSH receptor, sodium-iodide symporter, and thyroperoxidase mRNA [69]. CIGLI induced brain tumor stem cell differentiation [70]. In cultures of metaplastic urothelial cells, differentiation markers were increased after treatment with TRO [71].

### 5.3. TZD Effects in Animal Studies

PIO prevented lung tumor development in carcinogen-induced mouse models [72]. In a similar manner, PIO protected rats against chemically-induced (diethylnitrosamine and acetylaminofluorene) hepatocarcinogenesis [73]. PPAR*γ* could play a tumor-promoting role in hepatoma, because expression is significantly reduced in hepatocellular carcinoma with poor prognosis [74]. A similar situation is seen in endometrium carcinoma, where benign lesions show strong PPAR*γ* immunoreactivity but malignant lesions low to absent PPAR*γ* expression [12]. Chemoprevention of endometrial cancer by ROSI was observed in PTEN heterozygous mice [75]. Increased PPAR*γ* expression was predominantly seen in less invasive oral squamous cancer [76]. Chemically-induced oral squamous carcinoma in rats was reduced by 40% through administration of PIO [77] and tongue carcinoma formation was reduced by 40% by TRO [78].

On the other hand, tumor-promoting effects of PIO were observed in the APC^min⁡^ murine colon cancer model [41]. Because tumor-promoting effects were not seen in all cancer models, a model-specific effect cannot be excluded. The complex and, in part, opposing effects of TZDs on cancer development and progression can be explained by their cell-specific and species-specific action (tumor cells versus tumor environment). Effects of TZDs on immune cells may be the reason for the tumor-promoting effect of PIO in the APC^min⁡^ mouse model and the reduced tumor growth in immune-compromised mice and in the azoxymethane-induced tumor model [79]. While PPAR*γ* activation may decrease proliferation of tumor cells, it may increase macrophage polarization towards the M2 phenotype (TAM) and induce anti-inflammatory effects, also mediated by PPAR*γ* activation (see Section 3)

### 5.4. Human Data

One phase II trial on prevention of lung, head, and neck carcinoma in 21 patients with oral leukoplakia using PIO has been completed. Fifteen patients showed partial responses, 2 stable disease and 4 patients had progressive disease (NCT00099021; http://www.cancer.gov/clinicaltrials/). Based on these promising results, another trial on prevention of lung cancer is recruiting patients (NCT00780234; http://www.cancer.gov/clinicaltrials/).

In human trials, no general correlation of the protective effect of PPAR*γ* expression against tumor progression and chemopreventive effects of TZDs was obvious. While a protective role of PPAR*γ* expression was postulated in breast tumors and TZDs also acted preventive on the development of breast cancer in humans, the chemopreventive effect on colon cancer was not consistent with a protective role of PPAR*γ* expression in tumor samples.

## 6. Combined Treatments of TZDs with Other Drug Compounds

### 6.1. *In Vitro* Studies

Several studies evaluated the effect of combined therapies with TZDs and other agents. A large variety of combinations of TZDs have been evaluated* in vitro*. The observed antitumor effects include cytotoxicity/decrease of cell viability, growth inhibition, and apoptosis (for overview see Table 3).

In combination treatment with RXR-*α* ligands, increased cellular differentiation was reported [80, 81]. Some combined therapies take advantage of the cross-talk of PPAR*γ* with other signaling pathways. For instance, the upregulation of PTEN by ROSI rendered hepatoma cells more sensitive to the action of 5-fluorouracil [82]. Based on the idea of cross-talk between the ERK and PPAR*γ* pathways, combinations of ERK inhibitors and PPAR*γ* agonists could be useful in tumors with deleterious elevation of PPAR*γ*. Experimental data corroborate such an idea: gefitinib and ROSI increased growth inhibition of lung cancer cells and increased PPAR*γ* and PTEN expression [83]. Herceptin, an antibody against the EGF-receptor HER2, sensitized breast cancer cells for the differentiating action of TRO [84].

### 6.2. Animal Studies

The following examples show that improved antitumor responses were also obtained* in vivo*: growth of lung carcinoma xenografts and of chemically-induced breast tumors was inhibited by a combination of ROSI and platinum-based compounds [85, 86]. ROSI in combination with suberoylanilidehydroxamic acid (SAHA) decreased progression of preinvasive lung cancer in a murine model by 77% [46]. Similarly, a combination of TRO and platinum-based compounds increased survival of mesothelioma-xenografted mice [87]. The combination of EFA and paclitaxel reduced the size of anaplasic thyroid carcinoma xenografts [88]. Progression of ovarian carcinoma xenografts was slower when a combination of CIGLI and cisplatin was administered. Synergistic effects were reduction of angiogenesis and increased proapoptotic effects [89]. Aerosolized budesonide and oral PIO decreased lung cancer mass by 90% in a benz(a)pyrene-induced murine lung cancer model [90].

### 6.3. Human Data

Phase II trials of combination with the COX-2 inhibitor rofecoxib and PIO were able to induce complete response, partial responses, or stable disease in 5/5 angiosarcoma, 1/1 hemangioendothelioma, 4/19 metastatic melanoma, 10/40 soft tissue sarcoma, and 4/14 glioma patients [59, 91, 92]. Combination of PIO with other chemostatic drugs induced one complete response and prolonged disease-free survival in 2 of 19 patients with advanced melanoma enrolled in this phase II trial [91]. These data suggest potential efficacy of TZDs combined with other compounds in melanoma. For further evaluation of comedication with TZDs in patients, a prospective phase I/II trial of PIO combined with lenalidomide, dexamethasone, and treosulfan (NCT01614301) is currently recruiting patients (http://www.cancer.gov/clinicaltrials/).

According to human trials, only soft tissue sarcoma and melanoma might be sensitive to combinations of TZDs and COX-2 inhibitors and TZDs in polytherapy, respectively.

## 7. Specific Role of TZDs in Differentiated Thyroid Carcinoma (DTC)

PPAR*γ* has a specific role in thyroid cancer because follicular thyroid cancer is the only known neoplasm to be associated with a PPAR*γ* fusion gene product [93]. PAX8/PPAR*γ* is expressed in 30–35% of follicular thyroid carcinoma and 2–13% of follicular adenomas [94]. This chimeric protein is the result of a genetic translocation between chromosomes 2 and 3 and can activate the PPAR*γ* response element and induce proliferation. The mutation acts both as a gain and loss of function mutant in thyroid cancer and determines thyroid tumor differentiation; in more aggressive tumors gain of function predominates [93].

**Figure 3:**
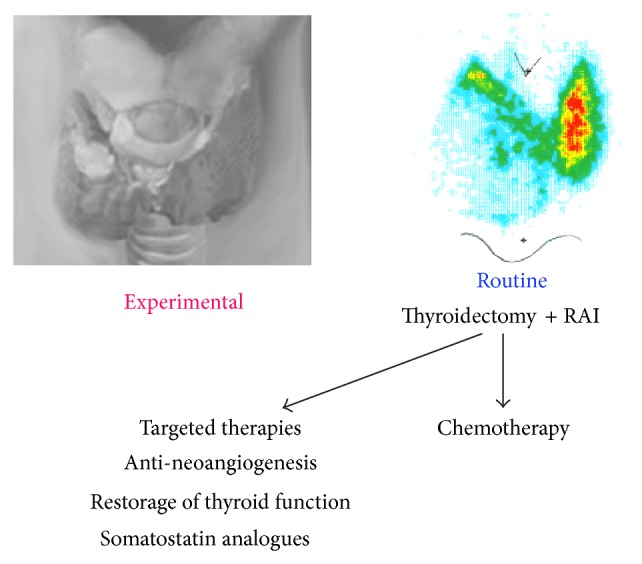
Overview of treatment options for DTC. Scheme of thyroid tumor (upper left) and scintigraphy with ^123^Iodide showing lack of uptake in the lower part of the right lobe (upper right).

Thyroid cancer incidence in the United States has increased in the last thirty years not only apparently because of enhanced detection but probably also as a true increase [95]. DTC is the most common type of thyroid carcinoma, mainly in the form of papillary thyroid carcinoma, accounting for 80–90% of all thyroid cancer cases. The second-most common form of DTC is follicular thyroid cancer with 10–15% incidence. The prognosis of DTC is generally good, with a 10-year survival rate of 85% [96]. A total of 10–20% of patients develops distant metastases [97]. In this group, the 10-year survival rate drops to 40%. Recurrence in DTC, however, occurs in up to a third of patients and only 30% of patients with distant metastases respond to radioiodine (RAI) therapy with complete remission [98, 99]. First-line treatment of DTC is by total or near total removal of the thyroid and if necessary lymph node dissection (Figure 3). This is generally followed by RAI treatment for thyroid remnant ablation and elimination of metastases. In case of insufficient efficacy of this treatment, doxorubicin is initiated [100]. Because doxorubicin treatment is not highly efficient, it is expected that, in the future, differentiating therapies will play a prominent role in cancer treatment. Redifferentiating compounds include retinoids, histone deacetylase inhibitors, DNA methyltransferase inhibitors, and TZDs. Somatostatin analogues such as ^68^Ga-DOTATOC are additional options for RAI-negative thyroid cancer [101].

PIO and CIGLI did not increase differentiation in a study on the human papillary carcinoma cell line NPA [102]. In another, TRO, ROSI, and PIO showed antiproliferative, proapoptotic, and differentiating effects on DTC cells [103]; TRO could increase expression of sodium-iodide symporter in DTC lines [104] and restore radioiodine-uptake* in vitro* [105].

### 7.1. Animal Studies

PIO was effective in reducing metastatic disease in a tumor model where the effect of PAX8/PPAR*γ* fusion protein is mimicked [106]. ROSI was also able to reduce thyrocyte growth by 40% in a murine knock-in model of thyroid hormone receptor *β* [107].

### 7.2. Human Data

In a small cohort of 5 patients treated with PIO for 6 months, no increase in RAI-uptake was seen [108]. Two case reports described successful induction of RAI-uptake after treatment with ROSI in a patient with noniodide avid metastases of DTC [109, 110]. Decreased thyroglobulin levels and tumor size indicated partial success of this treatment. Evidence for increased RAI-uptake upon treatment with ROSI was obtained in one of five patients enrolled in a pilot study [111]. In another pilot study, ROSI treatment resulted in positive RAI scans in 4/10 patients and a clinical trial showed increased RAI-uptake in therapeutic ^131^I scans in 5/23 patients [112, 113]. Despite reinduction of RAI-uptake in 5/20 patients of another phase II trial, none had a complete or partial response to ROSI after 3 months [114] by RECIST criteria [115]. The status of a current trial (NCT00098852) with ROSI for reinduction of radioiodine-uptake is not yet known (http://www.clinicaltrial.gov/). Also the redifferentiating action of PIO is being reassessed in a trial focused on follicular variants of PTC (NCT01655719; http://www.clinicaltrial.gov/). Interpretation of the results is complicated by limited accuracy of the technique of ^131^I scans and unknown status of receptor expression of the treated tumors, too low levels of expression by the target cells, inhomogeneity of RAI-uptake into the tumor, and the generally poor correlation between RAI-uptake and clinical remission, all of which may be reasons for lack of efficacy. In addition, observation time of less than one year may not be enough to monitor effects in slow-growing DTC.

## 8. Conclusion

Current data do not suggest a correlation of clinical efficacy and high PPAR*γ* expression according to mRNA and protein expression in tumor samples. This lack of relation could be due to methodical problems of PPAR*γ* detection in archived tumor samples and in the complexity of TZD action. First, TZDs show a variety of genomic and nongenomic effects and several antitumor effects occur independent of PPAR*γ*. This is particularly obvious in experiments where combination of PPAR*γ* agonists and antagonists act synergistically on inhibition of proliferation [116]. Cell specific effects of TZDs are particularly important in cancer because their action on immune cells may antagonize their effects on tumor cells. This suggests that administration of TZDs after tumor initiation may be inefficient or even deleterious and could explain why cancer mortality was increased in the meta-analysis of cancer incidence in patients with TZD treatment. Species-specific action was reported between human and murine endothelial cells where increase of proliferation was seen in the mouse cells and an antiproliferative effect in human cells [117]. Furthermore, TZDs show compound-specificity. TRO and CIGLI acted as antiproliferatives on ovarian cancer cell lines, while ROSI and PIO did not. This could be due to additional targets and/or PPAR*γ* independent effects; TRO for instance has stronger Akt/mTOR activity than the other TZDs. Finally, the effect of TZDs is concentration-dependent. Low concentrations of TZDs induced cell cycle arrest, while higher doses (>100 *µ*M) caused apoptosis. Effects at higher concentrations can be explained by transactivation of PPAR*γ* by cross-talk between signaling pathways where one receptor activates a receptor for a different ligand. Alternatively, TZDs may activate a specific subunit within a receptor oligomer [118]. As to the concentration, other coactivators may be involved in the effect and different downstream processes may be activated. PPAR*γ* agonists can also change the cell's expression of PPAR*γ* to different extents.

Against the background of limitations of traditional as well as new (transgenic) mouse models [119, 120] for human cancer, only efficacy in human trials is included in our final assessment. Use of TZDs in cancer might be therapeutic in prostate cancer and glioma, chemopreventive in colon, lung, and breast cancer, and increase therapeutic efficacy combined with other therapies in thyroid cancer, soft tissue sarcoma, and melanoma.

## Figures and Tables

**Table 1:** Relationship between protective role of PPAR*γ* expression and efficacy of TZDs in therapy.

Cancer type	Role of PPAR*γ*	TZD	Experimental model	Result	Reference
		PIO	Xenograft (HT-29) in mice with APC mutation, sc	Increased tumor growth	[41]
Colon	⇓/⇑		Azoxymethane-induced murine tumors	Reduced tumor growth	[39]
		TRO	HT-29 xenografts, sc	Reduced tumor growth and metastasis	[40]
			Metastatic colon cancer, 25 patients	All progressive disease	[43]

Lung	⇓/⇑	ROSI	Chemically-induced mouse model	Decrease in adenoma formation	[46]

		ROSI	LMM3 injection into mice, sc	Decreased tumor growth	[48]
Breast	⇓		Chemically induced rat model	Decreased tumor growth and incidence	[49]
		TRO	Advanced chemotherapy breast refractory cancer, 22 patients	No CR or PR, 3 SD	[50]
		ROSI	Early stage breast cancer, 38 patients	No decrease in proliferation	[51]

		PIO	PC3 xenografts, sc.	Decrease of bone-invasive potential	[53]
Prostate	⇓	TRO	Advanced prostate carcinoma, 41 patients	Stabilization of PSA levels	[54]
		ROSI	Recurrent prostate carcinoma, 1 patient	Delayed increase of PSA levels	[55]

Glioma	⟺	PIO	LN229 orthotopic xenografts	Reduced tumor volume, invasion	[58]
			Chemorefractory glioma, 14 patients	Disease stabilization (29%)	[59]

Melanoma	⟺	CIGLI	A375 xenografts, sc.	Growth inhibition, pro-apoptotic effects	[62]

		PIO	Transgenic mouse model (PPAR fusion protein/PTEN deletion)	Decreased tumor growth and metastasis	[106]
Thyroid	⇓	ROSI	Transgenic mouse model (Thyroid hormone receptor-*β* negative)	Delayed progression	[107]
			Metastatic thyroid cancer, 1 patient	Decrease in metastasis size	[109]

PPAR*γ* expression on tumor progression: promotion: ⇑; protection: ⇓; no effect: ⟺; CR: complete response; PR: partial response; SD: stable disease; sc: subcutaneous implantation of tumor cells.

**Table 2:** Summary of data on chemopreventive effects of TZDs in animal and human epidemiological studies.

Cancer type	Role of PPAR*γ* expression	TZD	Experimental model	Result	Reference
		PIO	Chemically-induced rat cancer model	Reduction of tumor incidence	[121]
Colon	⇓/⇑		Transgenic murine cancer model (nonsense mutation in the adenomatous polyposis coli)	Increase of tumor incidence	[122]
		TRO	Chemically-induced rat cancer model	Reduction of tumor incidence	[123]
		ROSI	Meta-analysis of diabetes trials	Reduced colon cancer incidence	[64]

Lung	⇓/⇑	PIO	Chemically induced murine cancer model	Reduction of tumor incidence	[72]
		PIO	Observational study	Reduced lung cancer incidence	[63]

Breast	⇓	PIO	Meta-analysis of diabetes trials	Reduced breast cancer incidence	[64]

Liver	⇓	PIO	Chemically induced rat cancer model	Reduced tumor incidence	[73]

Endometrium	⇓	ROSI	Transgenic murine cancer model	Reduced tumor incidence	[12]

Oral (squamous cancer)	⇓	PIO	Transgenic rat cancer model	Reduced tumor incidence	[77]
		TRO	Chemically induced rat cancer model	Reduced tumor incidence	[78]

PPAR*γ* expression on tumor progression: promotion: ⇑; protection: ⇓.

**Table 3:** Results of therapies combining TZDs with other antitumor treatments.

TZD	Additional compound	Model	Effect	Reference
	Gamma-radiation	Lung carcinoma cell lines (A549, H460)	DNA damage, apoptosis	[124]
	RXR-*α* ligands (SR11237, 6-OH-11-O-hydroxyphenanthrene)	Breast carcinoma cell line (MDA-MB231), lung carcinoma cell line (Calu-3), glioblastoma cell line (U87MG), melanoma cell line (G361)	Growth inhibition; apoptosis	[125–127]
CIGLI	TNF-*α*-related apoptosis inducing ligand	Ovarian cancer cell line (HEY)	Decrease of proliferation	[128]
	Lovastatin	Pancreatic carcinoma cell lines (Panc02, MIA, PACa-2), breast carcinoma cell lines (EMT6, MDA-MB-316), colon cancer cell line (C26)	Decrease of cell viability; decrease of proliferation	[129]
	Phenylbutyrate	Lung carcinoma cell lines (A549, H157)	Growth inhibition	[130]

	9-cis retinoic acid	Gastric carcinoma cell line (SGC7901)	Apoptosis	[131]
	Cisplatin	Lung cancer cell lines (A549, H522); mesotheloma cell line (EHMES-10)	Growth inhibition	[87, 132]
	Paclitaxel	Lung carcinoma cell lines (A549, H522)	Growth inhibition	[132]
	RXR-*α* ligands (bexarotene, all-trans retinoic acid)	Breast cancer cell lines (MCF-7, T-47D, ZR-75-1)	Growth inhibition	[133]
TRO	Cell signalling molecules (TRAIL, heregulin)	Ovarian cancer cell line (HEY); breast cancer cell lines (MCF-7, SKBR-3, MDA-MB-453)	Decrease of cell number; apoptosis	[128, 134]
	Lovastatin	Glioblastoma cell line (DBTRG05MG), lung cancer cell line (CL1-0)	Cell cycle inhibitor expression	[135]
	Aspirin	Lung cancer cell lines (CL1-0, A549)	Decrease of proliferation	[136]
	Tamoxifen	Breast cancer cell line (MCF-7)	Growth inhibition	[137]
	X-rays	Cervix cancer cell lines (HeLa, Me180)	Decrease of cell viability	[138]

	Platinium-based compounds (cisplatin, carboplatin)	Ovarian cancer cell lines (OVCA420, OVCA429, ES), lung cancer cell lines (A549, Calu-1, H23, H596, H1650)	Growth inhibition	[85]
	5-Fluorouracil	Hepatoma cell lines (BEL7402, Huh-7); colon cancer cell line (HT-29)	Decrease of cell viability, apoptosis	[82, 139]
	RXR-*α* ligands (bexarotene, 9-cis retinoic acid)	Breast cancer cell lines (MCF-7TR1, SKBR-3, T47D), colon cancer cell line (Moser)	Increase of differentiation, growth inhibition; decrease of cell viability	[80, 140]
ROSI	Cell signalling molecules (TNF-*α*, anti-Fas IgM, Seliciclib)	Breast cancer cell line (MDA-MB-231)	Growth inhibition	[141]
	Gemcitabine	Pancreas cancer cell lines (PANC-1, Panc02)	Decrease of cell viability, growth inhibition	[142]
	Gefitinib	Lung cancer cell line (A549)	Growth inhibition	[83]
	Herceptin	Breast cancer cell line (MCF-7)	Growth inhibition	[84]
	Bortezomib	Melanoma cell lines (MV3, FemX-1, G361)	Growth inhibition	[143]

	Paclitaxel	Lung cancer cell lines (A549, H522)	Growth inhibition	[132]
	RXR-*α* ligands (LG268)	Liposarcoma cells (primary)	Increase of differentiation	[81]
PIO	Statins (Simvastin, lovastatin)	Glioblastoma cell lines (U87, U138, LN405, RGII); meningeoma cell lines (IOMM-Lee, KT21-MG1)	Decrease of cell viability	[144, 145]
	Gemcitabine	Pancreas cancer cell line (PANC-1)	Decrease of cell viability	[142]
	2-Deoxyglucose	Prostate cancer cell lines (PC-3, LNCaP)	Decrease in tumor spheroid formation	[146]

EFA	Paclitaxel	Anaplastic thyroid carcinoma cell lines (DRO, BHT-101, ARO)	Growth inhibition	[88]
